# Leaf Trait Divergence and Elevational Adaptation in Endangered *Fagus hayatae*: Conservation Insights for an East Asian Paleoendemic

**DOI:** 10.1002/ece3.72185

**Published:** 2025-09-21

**Authors:** Ting Pan, Qian Yang, Hong‐yan Han, Xiao‐juan Liu, Meng‐xing Jia, Xiao‐hong Gan

**Affiliations:** ^1^ Key Laboratory of Southwest China Wildlife Resources Conservation, Ministry of Education Nanchong Sichuan People's Republic of China; ^2^ School of Life Sciences China West Normal University Nanchong Sichuan People's Republic of China

**Keywords:** elevational adaptation, *Fagus hayatae*, leaf functional traits, sympatric tree species

## Abstract

*Fagus hayatae* Palib. ex Hayata, an endangered East Asian paleoendemic species, dominates in Sichuan's Micang Mountain while persisting in fragmented populations across Taiwan and mainland China. Nevertheless, niche differentiation and adaptive strategies between this species and its close congener *(F. engleriana*) remain poorly understood. Leaf functional traits—key indicators of plant resource‐use strategies—reveal ecological adaptations through their interrelationships and environmental responses. Here, we examined interspecific variation in leaf functional traits among *F. hayatae*, *F. engleriana*, and co‐dominant tree species, and analyzed the trait variation in *F. hayatae* with respect to elevation to elucidate climate adaptation mechanisms (Micang Mountain Nature Reserve, China). *F. hayatae* and *F. engleriana* exhibited low niche overlap as community dominants. Compared to associated dominants, *F. hayatae* displayed higher leaf dry matter content (LDMC), specific leaf area (SLA), leaf carbon content (LCC), and leaf phosphorus content (LPC), alongside smaller stomata with lower density. Conversely, *F. engleriana* manifested stronger resource‐acquisition traits: larger leaf width (LW), leaf area (LA), SLA, LCC, higher leaf nitrogen content (LNC), and greater stomatal density, yet lower LPC. Direct comparison revealed that *F. hayatae* possesses larger but sparser stomata and reduced resource‐acquisition capacity relative to *F. engleriana*, evidenced by lower LL, LW, LA, SLA, LCC, and LNC. Elevation significantly modulated *F. hayatae*'s leaf traits. At lower elevations, increased LA and SLA indicated acquisitive tendencies, whereas higher elevations favored conservative strategies: reduced LA, SLA, stomatal area (SA), and LPC. This underscores *F. hayatae*'s superior phenotypic plasticity and reliance on defense‐oriented adaptations compared to sympatric *F. engleriana*. Critically, mid‐elevations supported optimal performance via coordinated stress‐tolerance traits. These findings highlight the priority of protecting mid‐elevation habitats where *F. hayatae* achieves peak fitness, and the need for proactive habitat preservation due to its conservative strategy, which entails slower post‐disturbance recovery.

## Graphical abstract

This study reveals how the endangered *Fraxinus hayatae* diverges from *F. engleriana* via stress‐tolerant leaf traits (e.g., stomatal morphology, resource allocation). Using elevational gradients in Micang Mountain, we demonstrate *F. hayatae*'s niche partitioning through coordinated plasticity in SLA, nutrients, and leaf anatomy. These evolutionary strategies inform conservation of paleoendemics under climate change.

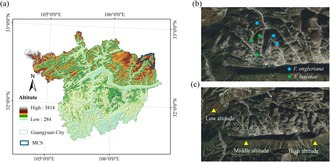

## 1 Introduction

Leaf functional traits—morphological and physiological adaptations to environmental conditions—serve as key indicators of plant ecological strategies along the Leaf Economics Spectrum (LES), a global axis ranging from acquisitive (fast resource capture) to conservative (stress‐tolerant) strategies (Wright et al. 2004). Comparative analysis of functional traits between closely related and sympatric species holds significant ecological implications (Li et al. 2025). These differences not only elucidate species coexistence mechanisms by revealing how functional trait divergence reduces competition and promotes niche complementarity, but also enable the assessment of different species' future adaptive potential through key trait comparisons positioned on the LES continuum, thereby providing a theoretical foundation for survival strategies and conservation measures (Holden and Cahill Jr. 2024). As the most environmentally responsive plant organs, leaves dynamically adjust their traits within this spectrum to enhance survival and performance across habitats (Chaturvedi et al. 2011). Critical LES trade‐offs include: acquisitive traits (e.g., high SLA, nutrient‐rich leaves) enhancing growth under resource abundance versus conservative traits (e.g., high LDMC, structurally reinforced tissues) ensuring persistence under stress. Studying these adaptations can contribute to revealing plant survival mechanisms and predicting their responses to environmental change across elevation gradients, where abiotic filters drive trait shifts along the LES.

Elevation gradients create natural environmental clines, with distinct variations in light, temperature, and precipitation, making them ideal for studying plant trait‐environment relationships. The “space‐for‐time substitution” approach leverages these gradients to predict how plant functional traits may respond to climate warming—a key methodology in global change biology (Walther et al. 2002; Kim and Donohue 2013). Existing research demonstrates species‐specific elevation adaptations: *Rhododendron* species exhibit differential thermal sensitivity in functional traits (Zhang et al. 2025; Pandey et al. 2024). *Castanopsis sclerophylla* showed altitude‐dependent trait shifts for cold adaptation (Liu et al. 2020). Such elevation‐mediated studies enable projections of plant performance under future climates, informing conservation strategies.

*Fagus hayatae* Palib. ex Hayata, an endemic deciduous tree to China, is sparsely distributed in mountainous areas of Sichuan, Hubei, Zhejiang, and northern Taiwan. As a National Grade II Protected Species, it faces habitat fragmentation and critically impaired regeneration, contrasting sharply with the stable sympatric congener *F. engleriana*. Given that leaf functional traits directly influence carbon assimilation and resource allocation—key determinants of seedling establishment and growth (Poorter et al. 2010)—this divergence may underlie their contrasting regeneration success (Li, Dong, et al. 2016; Li, Wu, et al. 2016). According to niche theory, functional trait divergence reduces interspecific competition by decreasing niche overlap, thereby enabling coexistence (Kraft et al. 2015). Consequently, the contrasting regeneration capacities of *F. hayatae* and *F. engleriana* may arise from divergent niche occupation—manifested through leaf trait differentiation. Furthermore, as climate warming further forces its distribution shift upward (Guo et al. 2014). However, the differences in leaf functional traits among *F. hayatae*, *F. engleriana*, and their sympatric species remain unclear, as do its elevational adaptation strategies.

This study compared leaf functional traits among *Fagus hayatae*, *Fagus engleriana*, and their dominant co‐occurring tree species, with a particular focus on the elevational adaptation strategies of *F. hayatae*. Our aims were to answer two questions: (i) Do interspecific differences in leaf functional traits underlie niche partitioning (quantified by niche breadth/overlap) and thereby drive divergent regeneration capacities between *F. hayatae* and *F. engleriana* ? (ii) What are the differences in leaf functional traits of *F. hayatae* at different altitudes, and are these differences the fundamental reasons for its varying adaptability to altitude?

## 2 Materials and Methods

### 2.1 Study Area

The investigation was conducted in Micang Mountain National Nature Reserve (32°29′–32°41′ N, 106°24′–106°39′ E), a biodiversity hotspot located along the subtropical‐temperate ecotone of the Micang Mountain–Daba Mountains in Sichuan Province. This region harbors mainland China's largest and first documented *F. hayatae* population (Chen 2014), which exhibits critical demographic vulnerabilities, including impaired regeneration and population collapse (Li, Dong, et al. 2016; Li, Wu, et al. 2016). Notably, this decline contrasts sharply with the stable populations of its sympatric congener, *F. engleriana*. Furthermore, as global warming degrades the cool‐humid habitats preferred by *F. hayatae*, larger individuals increasingly persist at higher elevations (Guo et al. 2014). This well‐preserved, expansive population provides an ideal system for comparative functional ecology studies. Characterized by a subtropical monsoon climate (mean annual temperature: 13.5°C–16.5°C; precipitation: 1100–1400 mm), the reserve exhibits pronounced altitudinal gradients that create distinct microclimatic conditions under the dual influence of southwest and southeast monsoons (Ying 1994; Liu et al. 2003). These elevational variations, combined with the sympatric distribution of two species, provide an exceptional natural laboratory for investigating leaf trait adaptations and assessing climate change impacts on relict tree species.

The *F. hayatae* forest forms a deciduous broadleaf community dominated by *F. hayatae* in the canopy, with associates including *Carpinus turczaninowii* and *Tsuga chinensis*. The understory features *Indocalamus tessellatus* and *Fargesia spathacea* thickets, while the herb layer contains characteristic species like *Carex tristachya* and *Tricyrtis macropoda*. The *F. engleriana* community similarly exhibits deciduous broadleaf dominance, with *Cornus kousa* subsp. *chinensis* and *Quercus shennongii* as main canopy associates. *Fargesia spathacea* and *Arundinaria fargesii* dominate the understory, accompanied by herbs such as *Rubia ovatifolia*.

### 2.2 Field Survey and Sampling

**FIGURE 1:**
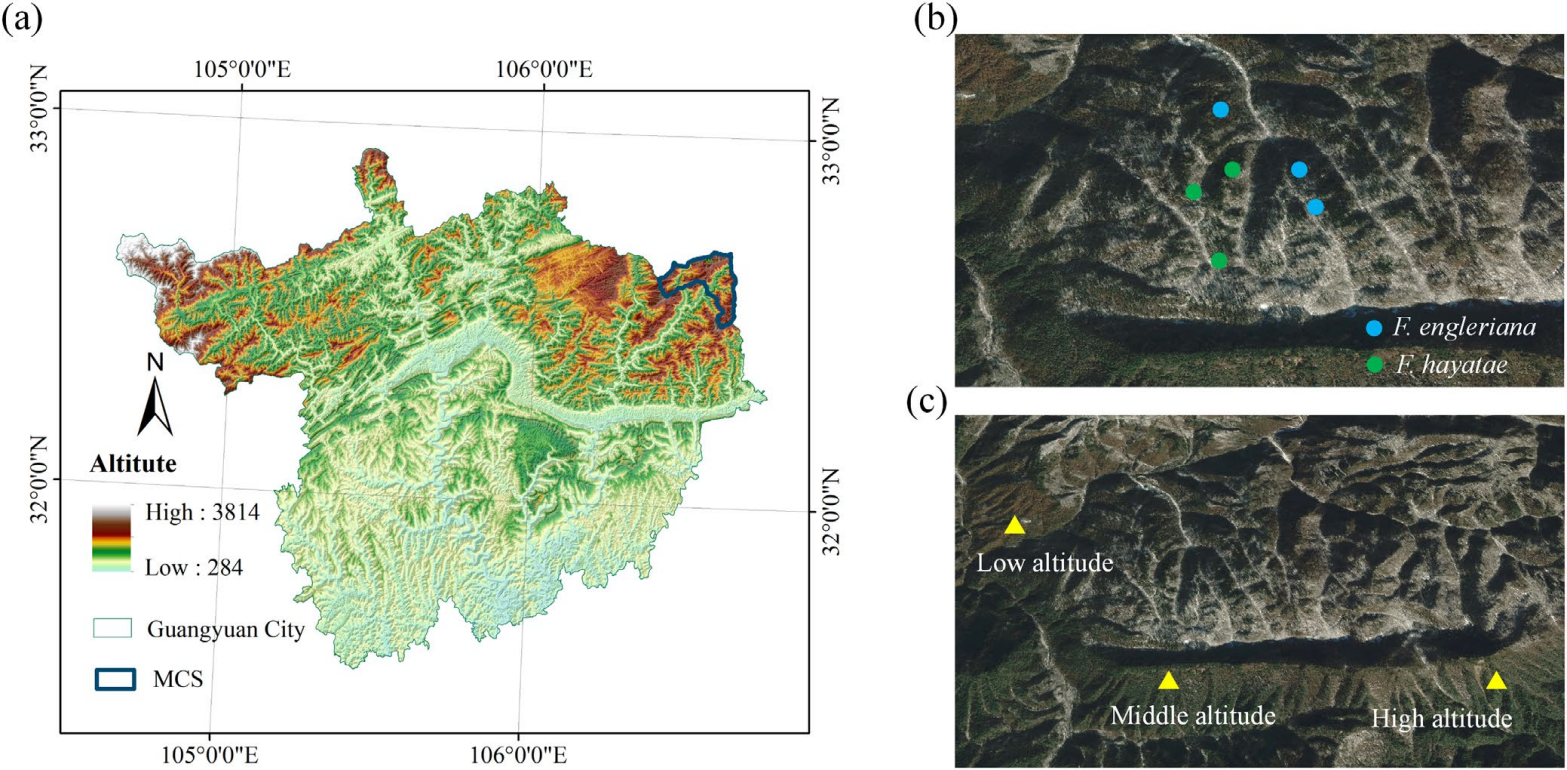
Schematic diagram of sample plot location. (a) geographical location of Micang Mountain Nature Reserve; (b) distribution of sampling sites for *Fagus hayatae* and *Fagus engleriana*; (c) distribution map of *F. hayatae* sampling sites at different altitudes.

During July 2022, we implemented a stratified sampling design to study *F. hayatae* and *F. engleriana* communities in the Reserve (Figure 1). To analyze interspecific dynamics, six 100 × 100 m primary plots—three per species—were established in minimally disturbed *F. hayatae* and *F. engleriana* stands. Each primary plot contained five systematically arranged 20 × 20 m subplots (30 subplots total) for canopy layer assessment.

To quantify elevational adaptation of *F. hayatae*, three discrete elevation bands were targeted based on documented microhabitat gradients (Badraghi et al. 2021; Wang, He, et al. 2016; Wang, Yu, et al. 2016): Low elevation (1654–1674 m), Mid elevation (1776–1790 m), and High elevation (1907–1931 m). One primary plot per band (total nine plots) was positioned on uniform slope aspects to isolate elevation effects from confounding factors (e.g., solar radiation, soil processes). This resulted in 45 subplots (5 per primary plot) systematically arranged within the elevation transect.

All plots were georeferenced with topographic parameters (slope, aspect) recorded. Following Xiang et al. (2015), every tree ≥ 5 cm DBH (*F. hayatae*, *F. engleriana*, and associates) was measured. Tree coordinates, slope gradient, aspect, height, and DBH were documented.

### 2.3 Sample Collection

Following quadrat analysis to determine species importance values, a standardized leaf sampling protocol was implemented for dominant *Fagus* species (*F. hayatae* and *F. engleriana*) and associated species with > 5% importance value (He et al. 2021). For each species, three healthy, similarly sized individual trees were randomly selected. From each tree, 3 outer‐canopy branches were collected using pole pruners, yielding 20–30 mature, intact, and healthy leaves per tree. Samples were immediately labeled and placed in resealable plastic bags. From the leaves collected per tree, nine intact leaves were selected for morphology and stomatal measurements (Table 1). These nine leaves were individually weighed for fresh weight (accuracy ±0.0001 g, Model CN‐FDC1003) and placed into separate, labeled tea bags. The remaining leaves were pooled and placed into 1–2 labeled tea bags per tree for subsequent measurement of leaf economic traits. All samples (individual and pooled tea bags) were preserved using rapid silica gel desiccation at room temperature and subsequently transported to the laboratory.

**TABLE 1:** Nineteen functional traits and descriptions.

Trait (abbreviation, unit)		Description
Morphological traits	Leaf length (LL, mm)	Distance from leaf base to apex; affects light interception area
	Leaf width (LW, mm)	Maximum leaf lamina width; influences light capture efficiency and gas diffusion resistance
	Leaf length:width ratio (LL/LW)	Leaf length divided by width; indicates shape and affects gas diffusion resistance/heat dissipation
	Leaf perimeter (LC, mm)	Total edge length of leaf lamina; reflects size and shape complexity
	Leaf Area (LA, cm^2^)	Surface area of a single leaf; determines light interception capacity
	Leaf thickness (LT, mm)	Vertical distance between epidermises; indicates structural tissue investment; correlates with stress tolerance and longevity
Stomatal Traits	Stomatal length (SL, μm)	Maximum pore length; affects maximum stomatal conductance and closure kinetics
	Stomatal width (SW, μm)	Maximum pore width; influences maximum aperture and gas diffusion conductance
	Stomatal L:W ratio (SL/SW)	Stomatal length divided by width; reflects morphology and responsiveness
	Stomatal perimeter (SC, μm)	Total border length of stomatal pore; used in conductance models
	Stomatal area (SA, μm^2^)	Surface area of open pore; directly determines maximum gas diffusion conductance (CO_2_/H_2_O)
	Stomatal density (SD, no./mm^2^)	Number per unit leaf area; high density often adapts to high light/dry conditions for finer regulation
Economic Traits	Leaf dry matter content (LDMC, mg/g)	Dry mass/water‐saturated fresh mass; core LES trait. High = dense tissue, conservative strategy, long lifespan, high stress tolerance, high potential WUE
	Specific leaf area (SLA, cm^2^/g)	Leaf area/dry mass; core LES trait. High = fast‐return strategy (low investment, high photosynthetic area), Low = slow‐return strategy (high investment, slow turnover)
	Leaf carbon content (LCC, mg/g)	Mass fraction of C in dry matter; mainly structural carbon (cellulose/lignin); relates to defense, support & decomposition
	Leaf nitrogen content (LNC, mg/g)	Mass fraction of N in dry matter; core LES trait, reflects photosynthetic enzymes/chlorophyll; indicates photosynthetic capacity & growth potential
	Leaf phosphorus content (LPC, mg/g)	Mass fraction of P in dry matter; indicates nucleic acids/phospholipids/ATP; reflects metabolic activity & growth (often limiting in low‐P environments)
	Carbon:nitrogen ratio (C:N)	Leaf C content/N content; indicates tissue quality, decomposition rate & N use efficiency; high = conservative nutrient strategy
	Nitrogen:phosphorus ratio (N:P)	Leaf N content/P content; key nutrient limitation indicator; high N:P suggests P limitation, low N:P suggests N limitation; reflects nutritional balance

### 2.4 Measurement of Leaf Functional Traits

#### 2.4.1 Morphological Traits

For the nine tea‐bag leaves per tree, the length (LL), width (LW), length‐to‐width ratio (LL/LW), perimeter (LC), and area (LA) of each leaf were measured using the Wanshen LA‐S series universal plant image analysis system (Microtek scanMaker i800plus). Leaf thickness (LT) was measured at three points (front, middle, and back) on the leaf, avoiding the midrib and secondary veins, using a digital vernier caliper with a precision of 0.01 mm. Each measurement was repeated three times, and the average value was taken as the LT.

#### 2.4.2 Stomatal Traits

The same nine tea‐bag leaves per tree were used for stomatal analysis. Leaves from each sample tree were cut into 1 × 1 cm pieces (excluding the ends for gymnosperms) at the midpoint. A dissociation solution was prepared by mixing equal parts of 20% nitric acid and 20% potassium dichromate. The leaf pieces were immersed in the dissociation solution for 24–48 h, depending on the plant species. After dissociation, the samples were washed in distilled water, the lower epidermis was carefully removed with tweezers, and the samples were fixed in FAA solution to prepare slides. The length (SL), width (SW), length‐to‐width ratio (SL/SW), perimeter (SC), area (SA), and density (SD) of stomata were measured using Motic Images Advanced 3.2 software.

Quality control: (1) SD: mean of three non‐overlapping fields/slide; (2) Morphology: ≥ 10 intact stomata/slide; (3) Validation: 15% slides (species‐stratified) re‐measured by a second observer. Relative errors (SD/SA) < 5%.

#### 2.4.3 Economic Traits

For the nine tea‐bag leaves per tree, were oven‐dried to constant weight at 80°C (Precision Oven, Model GZX‐9030MBE) and weighed using an analytical balance (accuracy ±0.0001 g, Model CN‐FDC1003) to measure leaf dry matter content (LDMC) and specific leaf area (SLA). The remaining dried leaves (11–21 per tree) were ground into a fine powder, sieved through an 80‐mesh screen to remove impurities, and analyzed for leaf carbon (LCC) and nitrogen (LNC) content using a VARIO Macro CN elemental analyzer. Leaf phosphorus content (LPC) was determined by digesting the samples with H_2_SO_4_‐H_2_O_2_, followed by colorimetric analysis at 700 nm using a UV spectrophotometer (UV‐4802) based on the molybdenum‐antimony resistance method and a regression equation.

### 2.5 Data Stratification and Analysis

#### 2.5.1 Community Characteristic Analysis

Importance Value (IV) Calculation:

Following the methods of previous studies (He et al. 2021; Zhang et al. 2022), this study selected associated species with an IV greater than 5% as the basis for comparing leaf functional traits of dominant species. The IV of tree layer species was calculated using the following formula:

(1) $$\mathrm{IV}=\frac{\mathrm{RF}+\mathrm{RA}+\mathrm{RD}}{3}\times 100\%$$

where IV is the importance value, RF is the relative frequency, RA is the relative abundance, and RD is the relative density.

#### 2.5.2 Niche Breadth

Given the uncertainty of resource availability and abundance within plots (Chen et al. 2020), this study used the Shannon‐Wiener niche breadth (BS) (Colwell and Futuyma 1971) and Levins niche breadth (BL) (Levins 1970) to comprehensively describe the niche breadth of the main canopy tree species in these communities. The calculation formula is as follows:

(2) $$B_{S}=-\sum_{j=1}^{r}p_{\mathrm{ij}}\text{In}\left(P_{\mathrm{ij}}\right)$$

(3) $$B_{L}=1/\sum_{j=1}^{r}{\left(p_{\mathrm{ij}}\right)}^{2}$$

where *r* is the total number of quadrats; *P*_*ij*_ *= n*_*ij*_ */N*_*i*_, *P*_*ij*_ represents the proportion of individuals of species *i* in resource state *j* to the total number of individuals of species *i*; *n*_*ij*_ is the quantity of species *i* utilizing resource state *j*, represented by the importance value of species *i* in quadrat *j* in this study; *N*_*i*_ is the total number of individuals of species *i*.

#### 2.5.3 Pianka Niche Overlap

The niche overlap index by Levins (Pianka 1973) was used to quantify the degree of niche overlap among species within communities. The calculation formula is as follows:

(4) $$O_{\mathrm{ik}}=\frac{\sum_{j=1}^{r}\left(n_{\mathrm{ij}}\times n_{\mathrm{kj}}\right)}{\sqrt{\sum_{j=1}^{r}n_{\mathrm{ij}}^{2}}+\sqrt{\sum_{j=1}^{r}n_{\mathrm{kj}}^{2}}}$$

where *O*_*ik*_ is the niche overlap value, *n*_*ij*_ and *n*_*kj*_ are the importance values (IV) of species *i* and *k* for resource *j*, respectively. *O*_*ik*_ ranges from 0 to 1, with values closer to 1 indicating higher overlap.

#### 2.5.4 Functional Traits and Their Altitudinal Adaptation

One‐Way ANOVA was conducted using IBM SPSS Statistics 23 to examine the differences in leaf functional traits among *F. hayatae*, *F. engleriana*, and their associated species, with Duncan's test for multiple comparisons. Independent samples *t*‐tests were used to analyze the significant differences in leaf functional traits between *F. hayatae* and *F. engleriana*, and SigmaPlot 14.0 was employed for graphical representation.

Pearson correlation analysis was applied to assess the relationships among leaf functional traits of *F. hayatae* at different altitudes, with correlation heatmaps generated using ggplot2 in R 4.1.3. Principal component analysis (PCA) of leaf functional traits in *F. hayatae* at different altitudes was performed using the psych package in R 4.1.3. Duncan's test was also used to examine the significant differences in leaf functional traits of *F. hayatae* at different altitudes.

## 3 Results

### 3.1 Ecological Characteristics of *F. hayatae* and *F. engleriana* Communities

#### 3.1.1 Importance Value and Niche Breadth of Canopy Trees

As shown in Table 2, the *F. hayatae* community consists of 17 canopy tree species, with five dominant species showing importance values exceeding 5%: *F. hayatae* (63.00%, clearly dominant), *Carpinus turczaninowii* (5.30%), *Tsuga chinensis* (5.24%), *Cornus kousa* subsp.*chinensis* (5.22%), and *Sorbus alnifolia* (5.11%). Niche breadth analysis using both Levins (BS) and Shannon‐Wiener (BL) indices revealed a consistent hierarchical pattern, with *F. hayatae* exhibiting the widest niche (BS = 1.093, BL = 14.348). The subdominant species showed progressively narrower niche breadths: *C. kousa* and *S. alnifolia* (BS = 1.055, BL = 11.778), followed by *T. chinensis* (BS = 1.011, BL = 9.000). These results demonstrated *F. hayatae's* superior competitive position and broad niche within the community.

**TABLE 2:** Important values in *F. hayatae and F. engleriana* communities.

Community	Species	RA (%)	RF (%)	RD (%)	IV (%)	Shannon‐Wiener (*B*_*S*_)	Levins (*B*_*L*_)
*Fagus hayatae*	*Fagus hayatae*	66.67	38.17	84.15	63	1.093	14.348
	*Carpinus turczaninowii*	5.51	4.96	5.42	5.3	1.011	7.571
	*Tsuga chinensis*	3.8	7.63	4.29	5.24	1.040	9.000
	*Cornus kousa* subsp.*chinensis*	4.76	7.63	3.28	5.22	1.055	11.778
	*Sorbus alnifolia*	4.76	4.96	5.6	5.11	1.055	11.778
*Fagus engleriana*	*Fagus engleriana*	58.72	31.65	74.75	55.04	2.693	14.549
	*Cornus kousa* subsp. *chinensis*	9.17	12.66	5.72	9.18	2.079	8.496
	*Carpinus turczaninowii*	7.34	10.44	5.89	7.89	1.792	6.850
	*Quercus shennongii*	4.59	6.33	4.33	5.08	1.386	4.774
	*Acer ceriferum*	2.75	6.33	0.85	3.31	0.693	2.334

The *F. engleriana* community comprises 18 canopy tree species, with four dominant species exhibiting importance values exceeding 5%: *F. engleriana* (55.04%, clearly dominant), *Cornus kousa* subsp. *chinensis* (9.18%), *Carpinus turczaninowii* (7.89%), and *Quercus shennongii* (5.08%). Niche breadth analysis revealed a consistent hierarchical pattern in both Levins (BS) and Shannon‐Wiener (BL) indices. *F. engleriana* demonstrated the widest niche (BS = 2.693, BL = 14.549), followed by *C. kousa* (BS = 2.079, BL = 8.496) and C. *turczaninowii* (BS = 1.792, BL = 6.850). *Sorbus alnifolia* (BS = 1.609, BL = 5.992) and *Q*. *shennongii* (BS = 1.386, BL = 4.774) showed progressively narrower niche breadths, confirming *F. engleriana*'s superior competitive dominance in this community.

#### 3.1.2 Niche Overlap of Canopy Layer in the Communities

**FIGURE 2:**
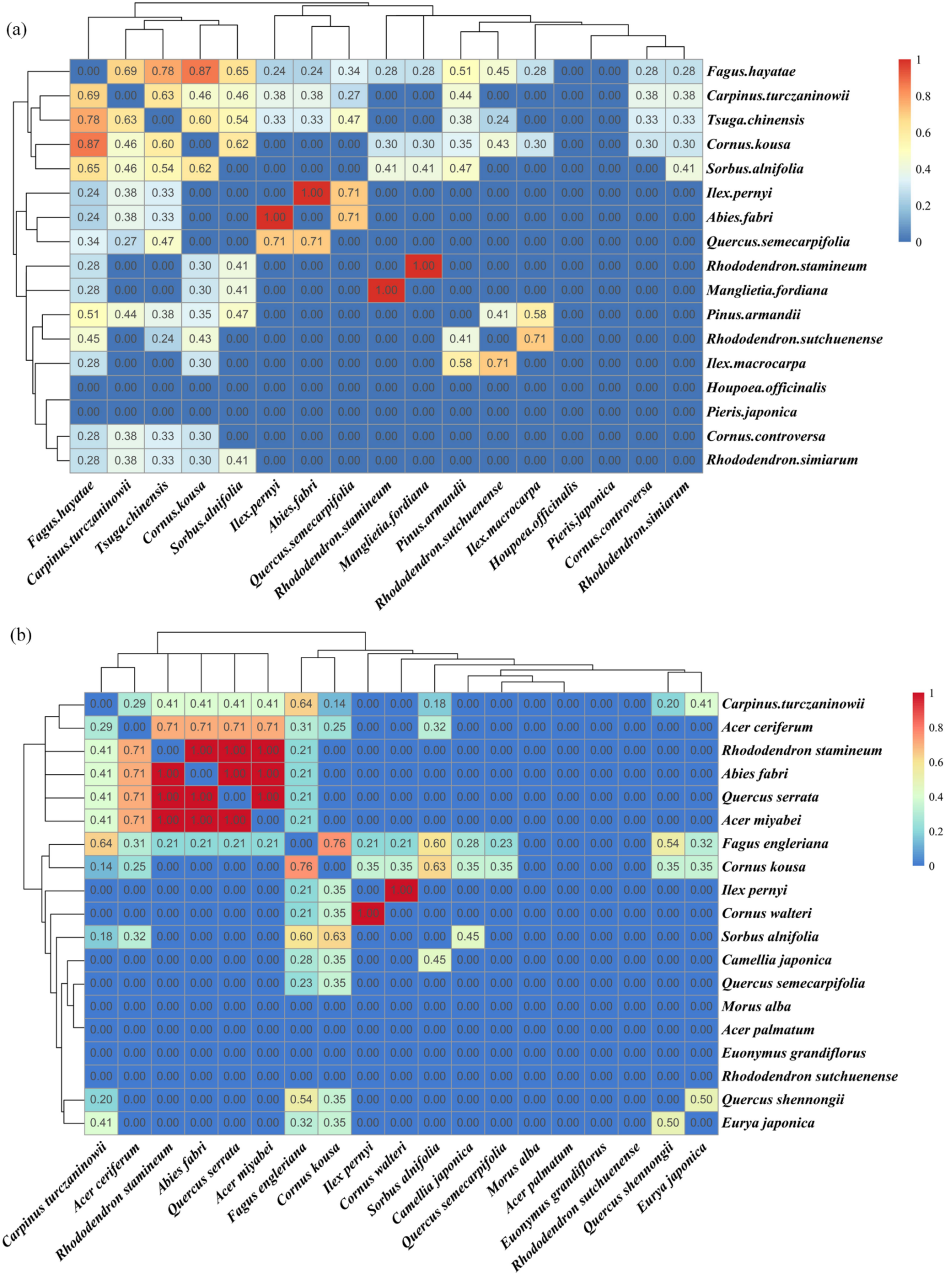
Niche overlap of main tree species in communities. (a) the niche breadth in *F. hayatae* communities; (b) the niche breadth in *F. engleriana* communities.

The analysis of niche overlap patterns in the canopy layer revealed important ecological relationships in the two *Fagus* communities (Figure 2). In the *F. hayatae* community, 31.25% of species pairs (5 out of 16) showed significant niche overlap (*O*_*ik*_ ≥ 0.50), with the strongest overlap occurring between *F. hayatae* and *Cornus kousa* subsp. *chinensis* (*O*_*ik*_ = 0.866), followed by *Tsuga chinensis* (0.784), *Carpinus turczaninowii* (0.693), *Sorbus alnifolia* (0.651), and *Pinus armandii* (0.506). Similarly, the *F. engleriana* community exhibited significant overlap in 22.22% of species pairs (4 out of 18), most notably with *C. kousa* (0.76), *C. turczaninowii* (0.64), *S. alnifolia* (0.60), and *Castanopsis sclerophylla* (0.54). Notably, both the two *Fagus* species demonstrated their highest niche overlap with *C. kousa* , suggesting potential competition for resources.

### 3.2 Comparative Analysis of Leaf Functional Traits

#### 3.2.1 Comparison of Leaf Functional Traits Between *Fagus hayatae* and Associated Species

**FIGURE 3:**
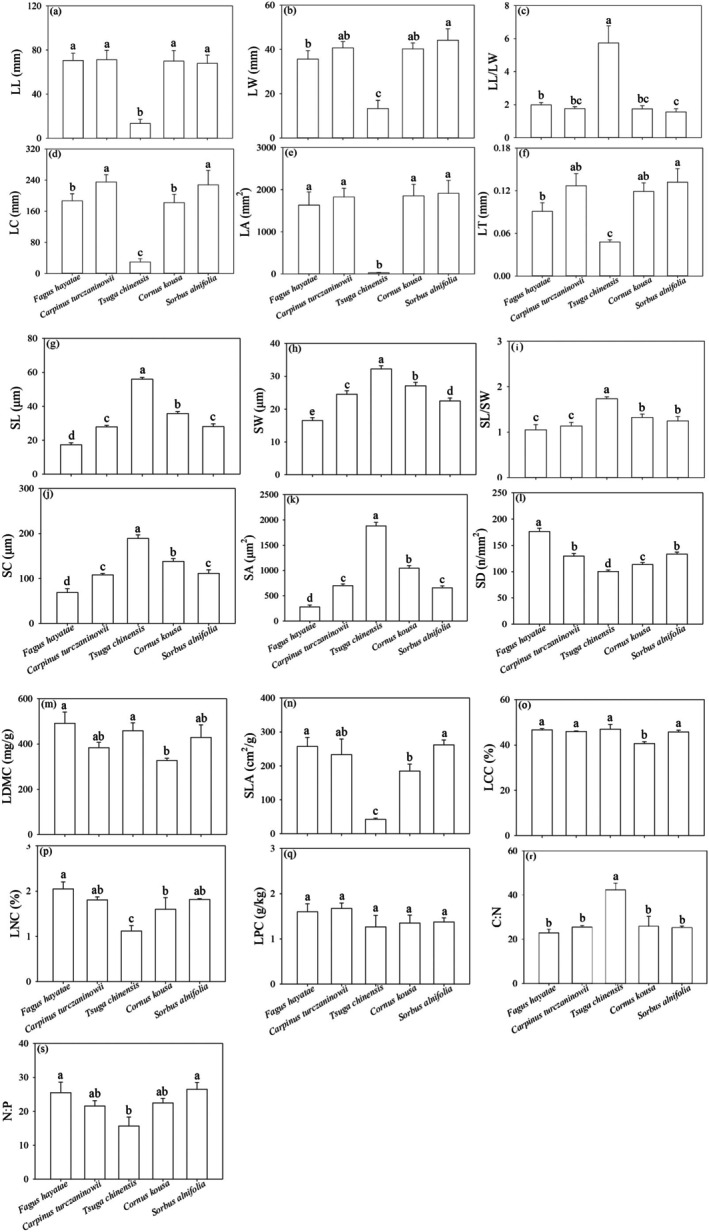
Comparison of leaf functional traits of *F. hayatae* and its dominant associated species. Leaf length (LL), (b) Leaf width (LW), (c) Leaf length to width ratio (LL/LW), (d) Leaf circumference (LC), (e) Leaf area (LA), (f) Leaf thickness (LT), (g) Stomatal Length (SL), (h) Stomatal Width (SW), (i) Stomatal length‐to‐width ratio (SL/SW), (j) Stomatal perimeter (SC), (k) Stomatal Area (SA), (l) Stomatal Density (SD), (m) Leaf dry matter content (LDMC), (n) Specific leaf area (SLA), (o) Leaf carbon content (LCC), (p) Leaf nitrogen content (LNC), (q) Leaf phosphorus content (LPC), (r) Carbon: Nitrogen Ratio (C:N), (s) Nitrogen: Phosphorus Ratio (N:P). Different letters above the bars indicate significant differences among species at *p* < 0.05. Error bars represent standard errors.

Comparative analysis of leaf functional traits revealed distinct ecological strategies between *F. hayatae* and its associated species (Figure 3). Morphologically, *F. hayatae* exhibited significantly larger leaf dimensions (LL, LW, LC, LA, LT; *p* < 0.05) than *Tsuga chinensis*, which displayed the most elongated leaves (highest LL/LW ratio). Stomatal characteristics showed *F. hayatae* possessed the smallest stomata (SL, SW, SC, SA) but the highest density (SD), while *Tsuga chinensis* had the largest stomata. Economic traits traits indicated *F. hayatae's* conservative resource strategy, with higher LDMC and LCC than *Cornus kousa* subsp. *chinensis* (*p* < 0.05), along with greater SLA and LNC compared to both *T. chinensis* and *C. kousa* (*p* < 0.05). Nutrient profiles showed significant differences in C:N and N:P ratios with *T. chinensis* (*p* < 0.05), while LPC remained similar across species (*p* > 0.05).

#### 3.2.2 Comparison of Leaf Functional Traits Between *Fagus engleriana* and Associated Species

**FIGURE 4:**
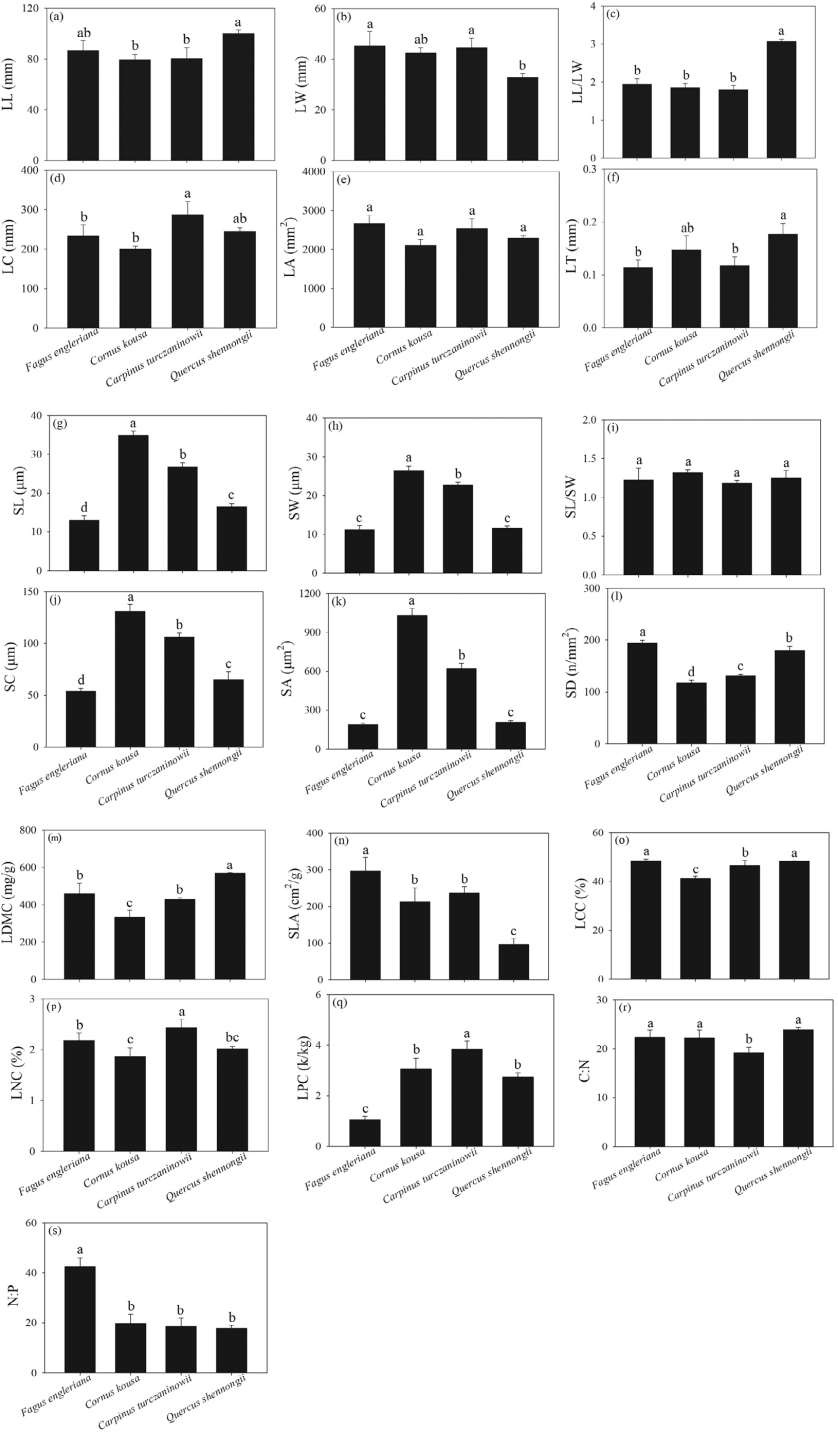
Comparison of leaf functional traits of *F. engleriana* and its dominant associated species. (a) Leaf length (LL), (b) Leaf width (LW), (c) Leaf length to width ratio (LL/LW), (d) Leaf circumference (LC), (e) Leaf area (LA), (f) Leaf thickness (LT), (g) Stomatal length (SL), (h) Stomatal width (SW), (i) Stomatal length‐to‐width ratio (SL/SW), (j) Stomatal perimeter (SC), (k) Stomatal area (SA), (l) Stomatal density (SD), (m) Leaf dry matter content (LDMC), (n) Specific leaf area (SLA), (o) Leaf carbon content (LCC), (p) Leaf nitrogen content (LNC), (q) Leaf phosphorus content (LPC). (r) Carbon: nitrogen ratio (C:N), (s) Nitrogen: phosphorus ratio (N:P). Different letters above the bars indicate significant differences among species at *p* < 0.05. Error bars represent standard errors.

Comparative analysis of leaf functional traits revealed distinct variations between *F. engleriana* and its dominant associated species (Figure 4). Morphologically, *F. engleriana* exhibited significantly greater leaf width (LW) but lower leaf thickness (LT) and length‐to‐width ratio (LL/LW) compared to *Quercus shennongii* (*p* < 0.05), while showing distinct leaf perimeter (LC) from *Carpinus turczaninowii* (*p* < 0.05). Stomatal characteristics demonstrated that *F. engleriana* possessed the smallest stomatal dimensions (SL, SW, SC, SA) but highest density (SD), contrasting with *Cornus kousa* subsp. *chinensis*, which had the largest stomata. Economic traits analyses showed *F. engleriana* had the highest specific leaf area (SLA) but intermediate leaf dry matter content (LDMC), with *Q. shennongii* exhibiting the opposite pattern (*p* < 0.05). Nutrient profiles revealed *F. engleriana* had the highest leaf carbon content (LCC), while *C. turczaninowii* showed the highest nitrogen (LNC) and phosphorus (LPC) contents (*p* < 0.05).

#### 3.2.3 Comparison of Leaf Functional Traits Between *Fagus hayatae* and *Fagus engleriana*

**FIGURE 5:**
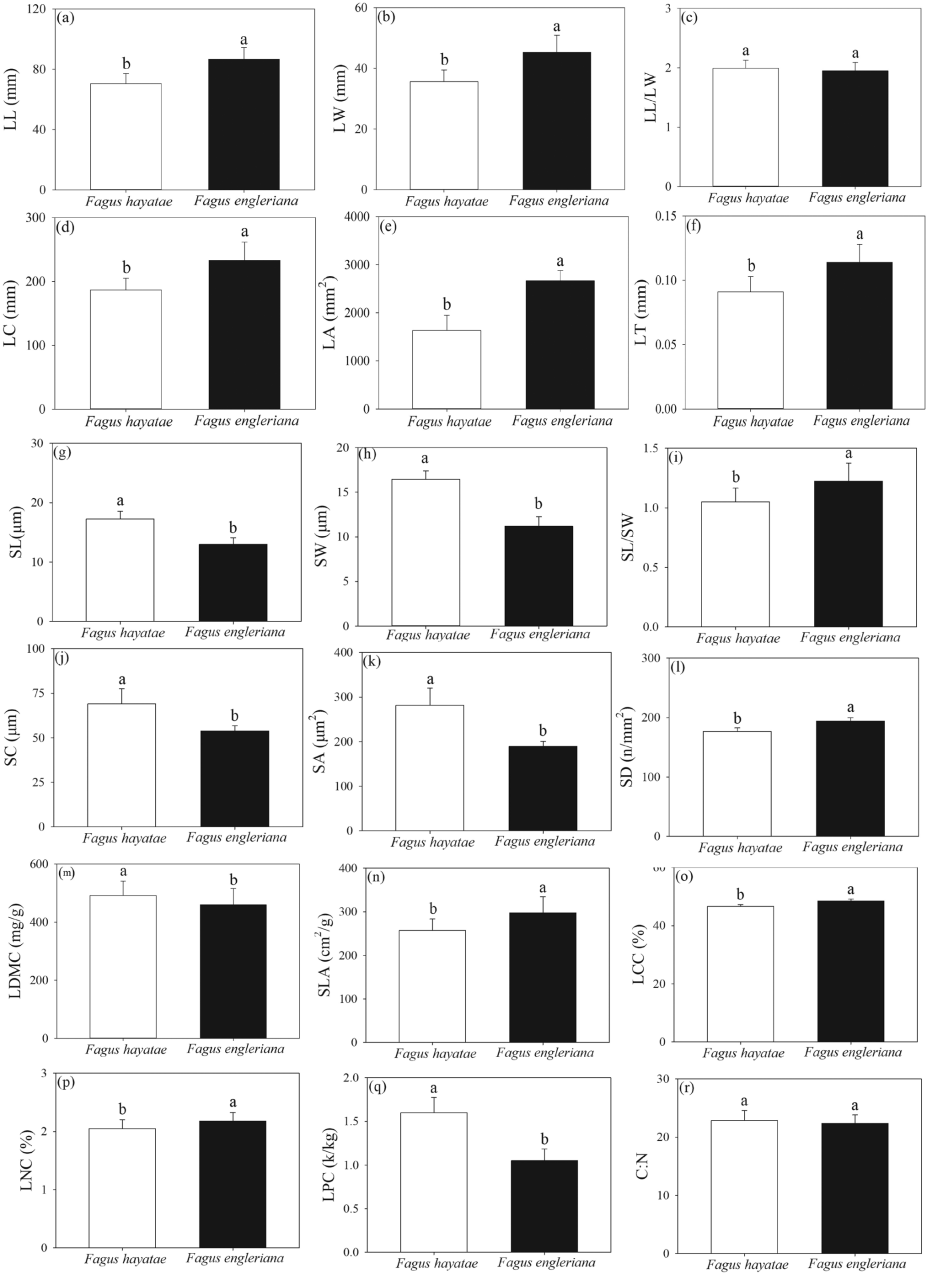
Comparison of leaf functional traits between *F. hayatae* and *F. engleriana*. (a) Leaf length (LL), (b) Leaf width (LW), (c) Leaf length‐to‐width ratio (LL/LW), (d) Leaf circumference (LC), (e) Leaf area (LA), (f) Leaf thickness (LT), (g) Stomatal length (SL), (h) Stomatal width (SW), (i) Stomatal length‐to‐width ratio (SL/SW), (j) Stomatal perimeter (SC), (k) Stomatal area (SA), (l) Stomatal density (SD), (m) Leaf dry matter content (LDMC), (n) Specific leaf area (SLA), (o) Leaf carbon content (LCC), (p) Leaf nitrogen content (LNC), (q) Leaf phosphorus content (LPC). (r) Carbon:Nitrogen ratio (C:N). Different letters above the bars indicate significant differences among species at *p* < 0.05. Error bars represent standard errors.

The comparative analysis revealed significant interspecific differentiation in leaf functional traits between *F. hayatae* and *F. engleriana* (Figure 5). *F. hayatae* exhibited a conservative resource‐use strategy characterized by significantly smaller leaf dimensions (LL, LW, LC, LA, LT; *p* < 0.05) yet larger stomatal structures (SL, SW, SC, SA; *p* < 0.05) compared to *F. engleriana*. Economic traits showed contrasting patterns, with *F. hayatae* possessing higher LDMC (*p* < 0.05) but lower SLA (*p* < 0.05), indicating thicker and denser leaves. Nutrient profiles differed markedly, as *F. hayatae* had significantly reduced LCC and LNC (*p* < 0.05) but elevated LPC (*p* < 0.05), resulting in a substantially lower N:P ratio (*p* < 0.05). The two species both maintained similar leaf allometry (LL/LW) and carbon‐nitrogen stoichiometry (C:N) (*p* > 0.05).

### 3.3 The Impact of Altitude on the Leaf Functional Traits of *Fagus hayatae*

#### 3.3.1 Correlation Analysis of Leaf Functional Traits

**FIGURE 6:**
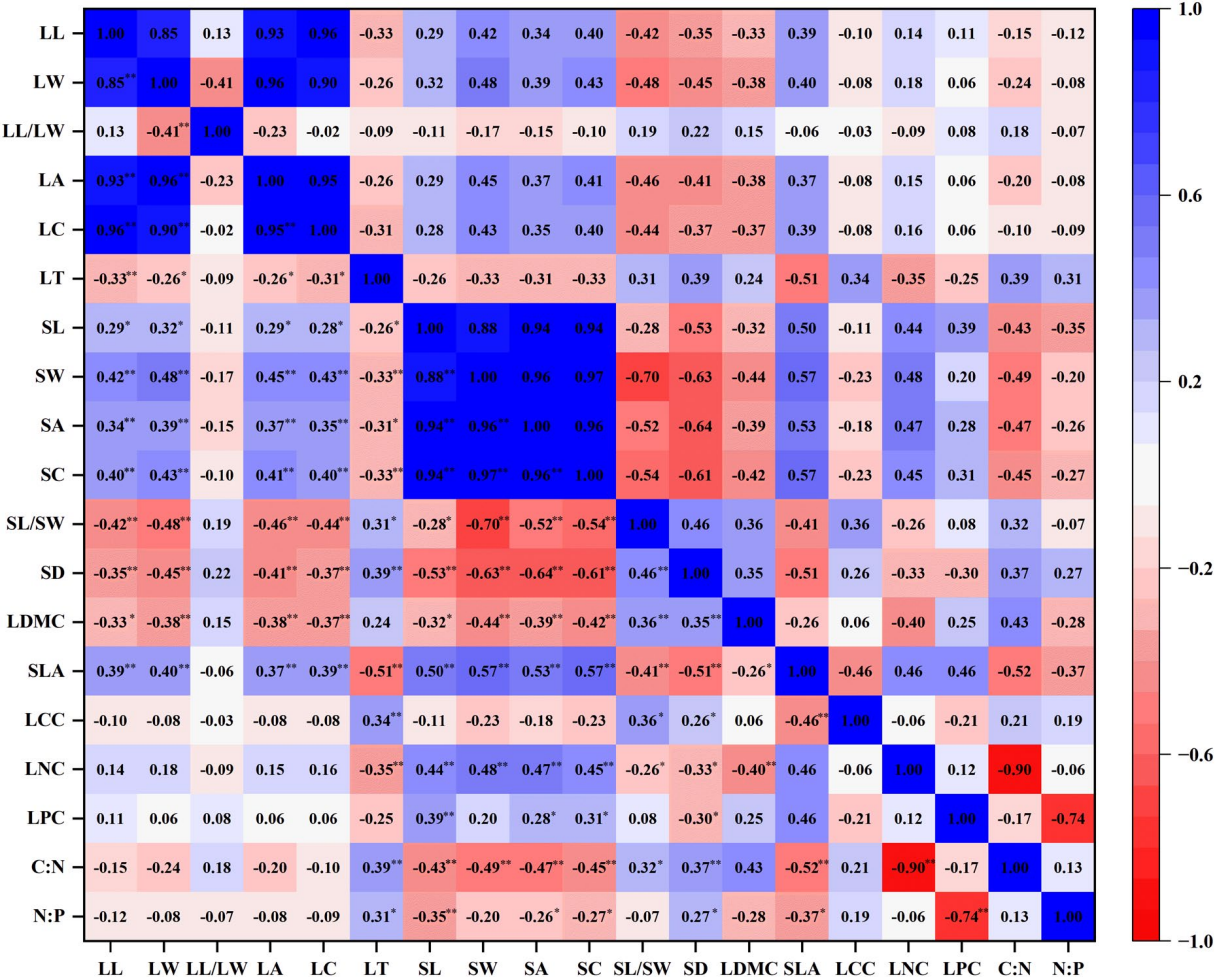
Correlation analysis of leaf functional traits of *F. hayatae* at different altitudes. *Represents a significant correlation at the 0.05 level, **represents a significant correlation at the 0.01 level.

Pearson correlation analysis revealed complex interdependencies among 19 leaf functional traits across elevational gradients (Figure 6). Key morphological traits (LL, LW, LA, LC) showed strong positive intercorrelations (*p* < 0.01), while exhibiting inverse relationships with structural traits (LT, SD, LDMC; *p* < 0.01). Stomatal characteristics demonstrated particularly notable patterns: SL correlated positively with SW, SA, and SC (*p* < 0.01), but negatively with SD and stoichiometric ratios (C:N, N:P; *p* < 0.01). The SL/SW ratio emerged as a key integrative parameter, showing strong positive associations with SD and LDMC (*p* < 0.01), but negative correlations with SLA (*p* < 0.01).

#### 3.3.2 Altitudinal Variation in Leaf Functional Traits: A Principal Component Analysis

**FIGURE 7:**
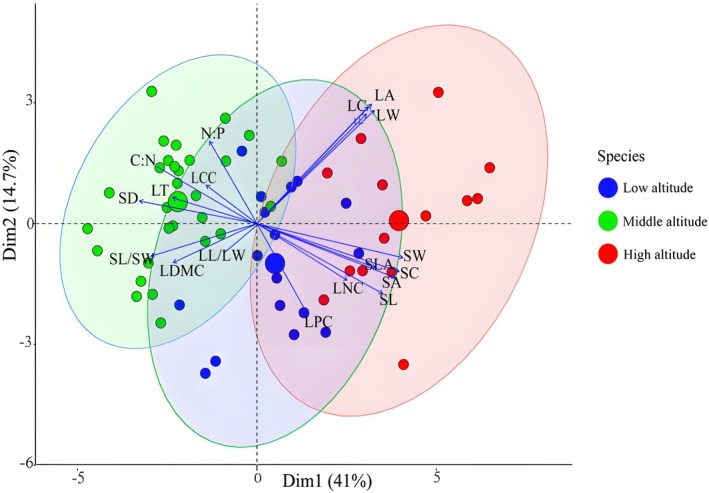
Principal component analysis of leaf functional traits of *F. hayatae* at different altitudes.

Principal component analysis (PCA) revealed distinct patterns in the leaf functional traits of *F. hayatae* across altitudinal gradients (Figure 7). While considerable overlap existed among elevations, trait differentiation was evident, with high‐altitude populations clustering along the positive PC1 axis (41.0% variance explained) and mid‐altitude populations along the negative PC1 axis. The first two principal components collectively accounted for 55.7% of total trait variation (PC1: 41.0%; PC2: 14.7%). PC1 was strongly influenced (loading > 0.5) by stomatal traits (SW, SC, SA, SL, SD) and leaf morphology (LW, LA, LC, LL, SLA). PC2 was primarily associated with leaf size (LA, LC, LW, LL), nutrient stoichiometry (LPC, C:N), and stomatal length (SL).

#### 3.3.3 Altitudinal Patterns in Leaf Functional Traits

**FIGURE 8:**
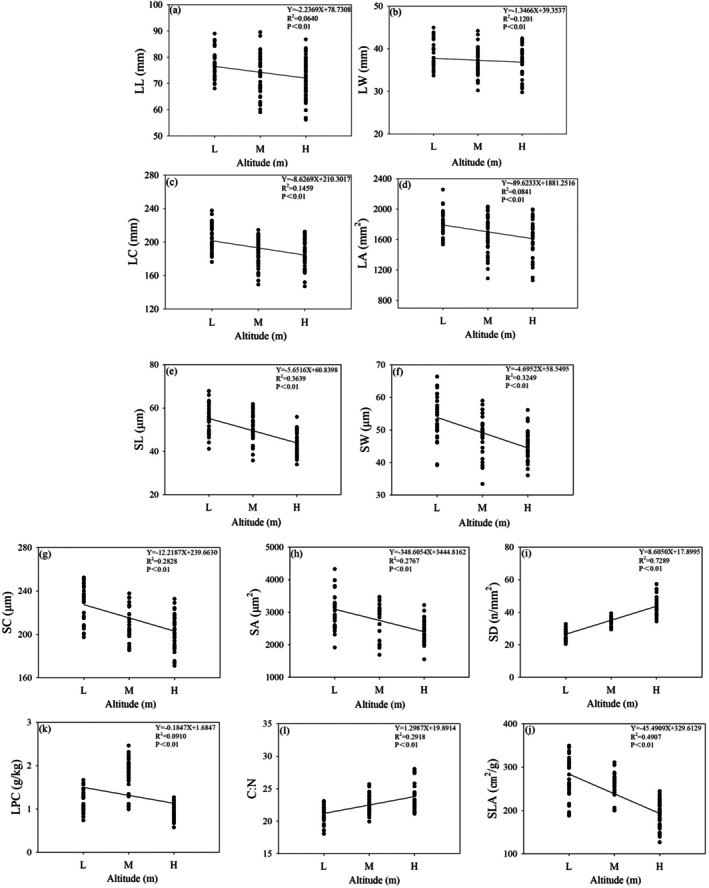
Variation of leaf functional characters of *F. hayatae* with increasing altitude. (a) Leaf length (LL), (b) Leaf width (LW), (c) Leaf circumference (LC), (d) Leaf area (LA), (e) Stomatal length (SL), (f) Stomatal width (SW), (g) Stomatal perimeter (SC), (h) Stomatal area (SA), (i) Stomatal density (SD), (j) Specific leaf area (SLA), (k) Leaf phosphorus content (LPC), (l) Carbon: nitrogen ratio (C:N). Different letters above the bars indicate significant differences among species at *p* < 0.05. Error bars represent standard errors.

Pearson correlation and linear regression analyses (Figure 8) revealed significant elevational trends in leaf traits of *F. hayatae*. Multiple morphological traits—including leaf dimensions (LL, LW, LC, LA) and stomatal characteristics (SL, SW, SC, SA)—showed strong negative correlations with elevation (*p* < 0.01), exhibiting progressive decreases with increasing altitude. Similarly, specific leaf area (SLA) and leaf phosphorus content (LPC) displayed significant altitudinal declines (*p* < 0.01), though LPC followed a unimodal pattern, initially increasing before decreasing at higher elevations. In contrast, stomatal density (SD) and carbon‐to‐nitrogen ratio (C:N) increased significantly with elevation (*p* < 0.01).

## 4 Discussion

### 4.1 Community Ecological Characteristics

The ecological dominance of tree species within the community can be effectively assessed through their importance values and niche breadths (Schellenberger et al. 2018). Our findings demonstrate that *Fagus hayatae* exhibited the highest importance value and niche breadth indices, confirming its status as the dominant species in this forest community. Subdominant species including *Carpinus turczaninowii*, *Cornus kousa* , *Tsuga chinensis*, and *Sorbus alnifolia* showed intermediate niche breadths, indicating their complementary ecological functions in the community.

The analysis of niche overlap patterns reveals a complex web of resource utilization strategies among canopy species (Costa‐Pereira et al. 2019) that most canopy species maintain differentiation (68.75% species pairs with Oik < 0.5), enhancing biodiversity through diversified resource strategies. This low‐overlap regime reduces competition and stabilizes communities, as predicted by coexistence theory (de Francesco et al. 2021). In contrast, the high niche overlap between *F. hayatae* and dominant co‐occurring species ( *Cornus kousa* , *Tsuga chinensis*) reflects convergent resource utilization strategies, triggering intense competition that depletes soil moisture and nutrients. Under such abiotic stress, seedlings accumulate reactive oxygen species (ROS) that disrupt metabolic processes and damage cellular structures. To mitigate ROS toxicity, plants activate antioxidant enzymes (e.g., SOD, CAT) and synthesize secondary metabolites (e.g., flavonoids), diverting energy from growth to defense (Bhusal et al. 2021). This physiological trade‐off explains the marked absence of seedlings and saplings in natural populations, ultimately crippling regeneration. This aligns with Li, Dong, et al. 2016; Li, Wu, et al. 2016), who found that as *F. hayatae* communities mature, seedlings require more nutrients and light, intensifying niche overlap with neighboring plants, overstory trees, and understory shrubs. This increased competition raises mortality rates and reduces survival.

What's more, Zhu et al. (2013) argued that endangered species in special habitats can exhibit population dominance within a community, but their population structure may not be healthy, and they still face difficulties in natural regeneration. Previous studies have shown that the *F. hayatae* population in the Reserve has a low number of existing seedlings and faces difficulties in natural regeneration (Li, Dong, et al. 2016; Li, Wu, et al. 2016). Therefore, a broad niche does not necessarily mean that *F. hayatae* is a generalist species; it may still face significant survival pressures, and population breeding efforts are urgently needed.

### 4.2 Adaptive Strategies of Leaf Function Traits in *F. hayatae* Compared to Dominant Associated Species

*F. hayatae* exhibits distinct functional traits that reflect its adaptive strategies in competition with dominant associated species. In leaf morphology, its comparable leaf area (LA) to most species (except *Tsuga chinensis*) suggests convergent light‐capture strategies under community competition (Xu et al. 2021), while its significantly lower leaf thickness (LT) than *Carpinus turczaninowii*, *Cornus kousa* , and *Sorbus alnifolia* indicates an “exploitative” growth strategy—prioritizing rapid expansion over nutrient storage (Xu et al. 2015). Thin leaves enhance light penetration to chloroplasts, boosting photosynthetic efficiency. Stomatal traits further support this strategy: *F. hayatae*'s small, high‐density stomata facilitate rapid environmental response and elevated photosynthetic capacity, contrasting sharply with the large stomata of the gymnosperm *T. chinensis* , which represent an adaptation for conservative water use (Lammertsma et al. 2011). Critically, this stomatal configuration enables fine‐tuned regulation of gas exchange, optimizing water‐use efficiency (WUE) under fluctuating environmental stresses as described in global stomatal function patterns (Wright et al. 2004).

Economic traits reveal *F. hayatae*'s competitive edge. Its higher specific leaf area (SLA) than *T. chinensis* and *C. kousa* aligns with efficient light harvesting and nutrient retention, while its superior leaf nitrogen content (LNC) underscores heightened photosynthetic capacity (Onoda et al. 2004). Stoichiometric ratios highlight phosphorus limitation (N:P > 16) in most species, suggesting *F. hayatae* thrives under moderate nitrogen availability (Güsewell 2004). Notably, its high leaf dry matter content (LDMC) indicates robust resource utilization and stress resilience, critical for dominance in heterogeneous environments.

In summary, *F. hayatae* combines rapid growth (via thin leaves, high SLA/LNC) with environmental responsiveness (small, dense stomata) and efficient resource use (high LDMC, balanced C:N), outcompeting associates through exploitative plasticity. This trait synergy enables persistence in light‐ and nutrient‐competitive niches.

### 4.3 Comparative Analysis of Leaf Functional Traits Between *F. hayatae* and *F. engleriana*

This study reveals key differences in leaf functional traits between *F*. hayatae and *F. engleriana*. In leaf morphology, *F. hayatae* has a smaller leaf area (LA), reducing light capture efficiency compared to *F. engleriana*, which maximizes photosynthetic gains through larger LA. Stomatal traits further support this: *F. hayatae* also exhibits larger, sparser stomata with slower response times, indicating weaker photosynthetic capacity and water‐use efficiency than *F. engleriana*.

Within the leaf economics spectrum, *F. hayatae* exhibits a low specific leaf area (SLA) and high leaf dry matter content (LDMC), reflecting a slow‐growth strategy that prioritizes long‐term storage over rapid resource acquisition (Grime et al. 1997). In contrast, *F. engleriana*, with its higher SLA, facilitates greater light interception per unit biomass and a faster carbon return on investment, enhancing its productivity and competitive dominance in resource‐rich environments (Wright et al. 2004; Wilson et al. 2010). Its elevated leaf nitrogen content (LNC) further supports this strategy by increasing the concentration of photosynthetic enzymes (e.g., Rubisco), directly boosting photosynthetic capacity and growth rates (Poorter et al. 2010; Hou et al. 2020). Stomatal traits also differentiate their adaptations: *F. hayatae*'s larger, sparser stomata exhibit slower response times to environmental fluctuations, reducing its ability to rapidly regulate water loss during drought stress and lowering water‐use efficiency (WUE). Inversely, *F. engleriana*'s smaller, denser stomata enable finer control over gas exchange, allowing quicker closure under water deficit to maintain hydraulic safety—a critical advantage in fluctuating or arid conditions (Lammertsma et al. 2011).

In summary, *F. hayatae* exhibits larger stomata, lower density, and reduced resource‐acquisition traits (via LL/LW/LA, SLA/LCC/LNC) versus *F. engleriana*, reflecting poorer resource efficiency, competitiveness, and adaptability. These synergistic trait deficiencies drive persistent declines in seedling establishment and sapling survival, aligning with IUCN criteria for endangered species designation.

### 4.4 The Impact of Altitude on the Leaf Functional Traits of *F. hayatae*

#### 4.4.1 Coordinated Adaptation of Functional Traits in *F*. *hayatae*

To decode the multivariate coordination of leaf functional traits and identify key adaptive trade‐offs across altitudes, we employed Principal Component Analysis (PCA) and correlation networks. Plants optimize functional trait combinations through trade‐offs to adapt to environmental constraints (Wright et al. 2007).

In *F*. *hayatae*, key trait correlations reveal coordinated strategies: LA shows a positive correlation with specific leaf area (SLA) but a negative correlation with leaf dry matter content (LDMC), reflecting a balance between light capture (high LA/SLA) and water‐use efficiency (high LDMC) (Shipley 1995; Fonseca et al. 2000). This aligns with global patterns, where thinner, larger leaves enhance metabolic rates in resource‐rich environments. Crucially, we observed a key trade‐off between stomatal density (SD) and stomatal size (SL, SA) (Wang, He, et al. 2016; Wang, Yu, et al. 2016). Higher SD, often associated with smaller stomata (Kardiman and Ræbild 2018), is linked to faster stomatal opening and closing kinetics, potentially enabling a rapid response to fluctuating environmental conditions such as sudden bursts of sunlight or vapor pressure deficit (Kardiman and Ræbild 2018). Concurrently, this stomatal morphology (SD vs. size) is intrinsically linked to water‐use efficiency (WUE). Generally, lower SD and larger stomatal size tend to correlate with higher intrinsic WUE (reflected in less negative leaf carbon isotope discrimination) by reducing cuticular and stomatal conductance (Petrík et al. 2023). In *F. hayatae*, stomatal density (SD) declines with increasing LA, likely due to mesophyll cell expansion reducing stomatal space per unit area. These coordinated adjustments in stomatal traits enable efficient gas exchange under varying altitudinal pressures: smaller, denser stomata potentially offering faster response times advantageous at high elevations for mitigating cold‐induced hydraulic stress and coping with shorter growing seasons, while larger, sparser stomata favor steady CO_2_ uptake and potentially higher WUE at lower elevations (Wang, He, et al. 2016; Wang, Yu, et al. 2016; Petrík et al. 2023). It is noteworthy that these relationships between stomatal anatomy may vary between juvenile and mature developmental stages (Petrík et al. 2024), highlighting the dynamic nature of trait optimization across the plant lifespan. The negative C:N vs. leaf nitrogen content (LNC) correlation highlights a carbon‐nitrogen trade‐off: higher carbon investment (e.g., structural compounds) reduces nitrogen availability for photosynthesis (Yan et al. 2024). Similarly, N:P ratios are driven by phosphorus (LPC), with high N:P (> 16) indicating phosphorus limitation, a common constraint in *F. hayatae*'s (Yang et al. 2010).

These trait networks allow *F. hayatae* to balance light acquisition (LA/SLA), water economy (LDMC/SD), and nutrient use (C:N/N:P) across altitudes. Smaller, thicker leaves with dense stomata dominate high‐elevation stress zones, where low SLA and high LDMC minimize water loss and frost damage (Wright et al. 2007), while larger leaves with optimized stomata and nutrient ratios enhance competitiveness at lower elevations. Such integrated adjustments underscore its niche differentiation within forest ecosystems.

#### 4.4.2 Altitudinal Adaptation of Leaf Functional Traits in *F*. *hayatae*

Plants and their environments are intricately linked, with morphological traits reflecting long‐term evolutionary adaptations to climatic conditions (Li et al. 2013). Altitudinal gradients impose dramatic shifts in light, temperature, humidity, and moisture, driving distinct patterns in plant functional traits. *F*. *hayatae* exhibits marked altitudinal variation in leaf morphology: leaf length (LL), width (LW), perimeter (LC), and area (LA) decrease with elevation, likely due to reduced interspecific competition at higher altitudes. Smaller leaves at high elevations minimize maintenance costs, mitigate frost damage by lowering saturated water content (Wright et al. 2007), and reduce wind resistance (Givnish et al. 1984). Stomatal traits of *F. hayatae* also shift with elevation. Stomatal size decreases at higher altitudes, enabling faster responses to fluctuating conditions like low temperatures and high UV‐B (Hetherington and Woodward 2003), while stomatal density (SD) increases to compensate for lower CO_2_ and O_2_ partial pressures (Mott et al. 1982; Liu et al. 2020) and enhance drought tolerance via rapid stomatal closure.

Specific leaf area (SLA) declines with elevation, reflecting a shift toward stress resistance: water conservation strategies under cold‐induced root uptake limitations (Hultine and Marshall 2000) and enhanced cold resistance. Nutrient allocation of *F. hayatae* follows a mid‐elevation peak: leaf phosphorus content (LPC) is highest at mid‐altitudes, where balanced conditions favor photosynthetic investment but declines at higher elevations as resources shift to protective tissues. Notably, N:P ratios consistently exceed 16 across elevations, indicating phosphorus‐limited growth—a pattern consistent with broader findings in Chinese vegetation (Ren et al. 2007; Koerselman and Meuleman 1996).

The studies showed that at low altitudes, plants increase LA and SLA to enhance light capture, while at high altitudes, they adopt a conservative strategy—reducing LA, SLA, SA, and LPC—to improve stress resistance, particularly against cold‐impaired photosynthesis and water stress. These trait variations underscore *F. hayatae*'s adaptive trade‐offs between growth efficiency and stress tolerance across altitudinal gradients, highlighting that high‐altitude regions are unfavorable for its growth and reproduction. Using a space‐for‐time substitution approach, it is inferred that under global warming, the fitness of *F. hayatae*'s leaf functional traits will decline, hindering population regeneration and recruitment. Consequently, *F. hayatae* is likely to shift its distribution toward higher elevations in the future.

## 5 Conclusion

Functional trait comparisons between *Fagus hayatae* and its sympatric species, *F. engleriana*, as well as across elevational gradients, reveal adaptive plasticity and critical regeneration constraints, guiding evidence‐based conservation efforts. Specifically: (Wright et al. 2004) Adaptive advantages over sympatric competitors include stress‐tolerant traits, such as elevated leaf dry matter content (LDMC) and leaf phosphorus concentration (LPC), and efficient stomatal regulation (characterized by smaller, denser stomata). These traits secure niche dominance in stable habitats. (Li et al. 2025) Regeneration impairment arises from conservative resource allocation compared to *F. engleriana*: reduced specific leaf area (SLA) and leaf nitrogen concentration (LNC) limit light and nitrogen acquisition, thereby crippling seedling competitiveness under interspecific competition. (Holden and Cahill Jr. 2024) Elevational plasticity peaks at mid‐elevations (1600–1900 m) with balanced trait coordination, exemplified by the SLA‐LDMC equilibrium. However, resource reallocation to defense at higher elevations, indicated by reduced leaf area (LA) to SLA ratios, exacerbates recruitment bottlenecks. To overcome these constraints, we propose the following management strategies: (Wright et al. 2004) Priority conservation of mid‐elevation core zones (1600–1900 m) as climate refugia; (Li et al. 2025) Phosphorus supplementation in regeneration microsites to alleviate N:P ratios greater than 16. Unfortunately, this study focused solely on leaf‐level traits, lacking assessments of key physiological processes, whole‐plant architecture, and soil properties. Future investigations that integrate these dimensions will enhance our understanding of *F. hayatae*'s environmental adaptations.

## Author Contributions

**Ting Pan:** conceptualization (equal), data curation (equal), investigation (equal), methodology (equal), software (equal), writing – original draft (equal). **Qian Yang:** data curation (equal), investigation (equal), software (equal), visualization (equal). **Hong‐yan Han:** funding acquisition (equal), methodology (equal), project administration (equal). **Xiao‐juan Liu:** investigation (equal), methodology (equal), resources (equal). **Meng‐xing Jia:** investigation (equal), methodology (equal), resources (equal). **Xiao‐hong Gan:** data curation (equal), funding acquisition (equal), methodology (equal), project administration (equal), resources (equal), supervision (equal), visualization (equal), writing – review and editing (equal).

## Conflicts of Interest

The authors declare no conflicts of interest.

## Supporting information


**Data S1:** ece372185‐sup‐0001‐Supinfo01.xlsx.

**Data S2:** ece372185‐sup‐0002‐Supinfo02.xlsx.

**Data S3:** ece372185‐sup‐0003‐Supinfo03.xlsx.

**Data S4:** ece372185‐sup‐0004‐Supinfo04.xlsx.

**Data S5:** ece372185‐sup‐0005‐Supinfo05.xlsx.

**Data S6:** ece372185‐sup‐0006‐Supinfo06.xlsx.

**Data S7:** ece372185‐sup‐0007‐Supinfo07.xlsx.

## Data Availability

All the required data is uploaded as Supporting Information.
